# Patriotism and the Nurse-Training Schools

**Published:** 1916-03-25

**Authors:** 


### PATRIOTISM AND THE NURSE-TRAINING SCHOOLS.

### VII. A Typical Instance of Good Organisation.

There are several hospitals which suffered a severe strain at the outbreak of war from the fact that their matron was also principal matron of the Territorial hospital for the district. It is interesting to observe that what might easily in weak hands have become a danger to the administration, has frequently turned to the advantage of both the military and civil institutions.

### Bristol Royal Infirmary.

No institution has risen more worthily to the occasion than this. No fewer than fifty-seven of the staff, including the matron, and over fifty former sisters and nurses are doing war work, while from fifteen to twenty of the probationers are working in the 2nd Southern General Hospital.

The outbreak of war in August 1914 found the Bristol Royal Infirmary ready to help. In accordance with the scheme arranged for by the governors at their annual meeting in 1910, and graciously acknowledged in His Majesty's speech at the opening of the King Edward Memorial Infirmary on June 28, 1912, this wing of the Royal Infirmary was at once handed over to the military authorities, and on August 12, 1914, 270 beds were available for wounded soldiers.

The matron of the Bristol Royal Infirmary is the principal matron of the 2nd Southern General Hospital. Many of the sisters and nurses are members of the Territorial Force Nursing Service, and were on the spot ready for work.

From August 5 Sister Vicary, Nurse Shaw, and Nurse Taylor left for work at the Royal Naval Hospital at Portland. Nurses Morrin, Timpson, and Barbier left for France. Nurse Barbier has been awarded the Royal Red Cross. Sister Wood, and Nurses Remnant, Blades, and Rapson left for France, and Nurses Bell, Owen, and Beaumont for service at home. Sister Hobbs and Nurse Pope worked for some time on a hospital ship, and are now in hospital at Aldershot and Netley. Miss Dent, the matron's assistant, is acting matron of the Royal Infirmary Section of the 2nd Southern General Hospital. Miss Hill, the matron's junior assistant, is assistant matron at the Southmead part of the 2nd Southern General Hospital.

Other Royal Infirmary sisters and nurses who have been called up for duty in the Territorial Force Nursing Service are :—Sisters Merry, Hobson, Blain, Hughes, Brander, K. I. Baker, Hutchinson, Shepherd, Kilburn, Peers, Molyneux, Adlem, Alexander, Ward; Nurses J. M. Creech, Hall, May, Hathway, Mee, Gould, F. M. Baker, Lister, Jackson, Stowell, Wills, Goodwin, S. A. Evans, V. G. Jones, E. E. Brown, Tyack, Pearce, Griffith, Lynch, Bowyer, Gibbs. Many of these have gone abroad on active service.

During the year, Sister Wilkin and Nurse Morton have left for work at the Royal Naval Hospital, Plymouth; Nurse Kingdom has gone to Haslar and Nurse Wilde to Egypt, while Nurse Stapleton is working in France.

The following former sisters and nurses are doing military work :—Miss White is matron of the 3rd Southern General Hospital at Oxford; Miss R. Osborne holds the post of matron in the Q.A.I.M.N.S. (Queen Alexandra's Imperial Military Nursing Service), and has been mentioned in dispatches; Miss S. Wooler is also a member of the Q.A.I.M.N.S., and is acting matron of a hospital ship. Miss Samson is at the Brook War Hospital.

Other sisters and nurses who have joined the Q.A.I.M.N.S. Reserve are Sister Bleazby and Nurse G. Kershaw, working in Malta; Nurse Dodd, working in Egypt; Sister Turner, working at the Beaufort War Hospital, Bristol; Sister Chapman, in France; and Sister Creech, at King George's Hospital. Sister Bradley and Nurse M. D. Evans are at the Epsom War Hospital, and Nurse Tarver at Chalons-sur-Marne.

The Matron of the Queen Victoria Memorial Hospital, Nice (which is now devoted to French wounded soldiers), Miss Bryant, was trained at the Bristol Royal Infirmary, and has with her Sister Hawkins and Nurse Derrick.

The following sisters and nurses are working in Red Cross Hospitals at home :—Sisters F. White, E. K. Webb, K. P. Jackson, Prout, Walker; Nurses G. Amos, Lambert, Jacomb-Hood, Fuller, Wesley, Stephens. Nurse Studley is in a Red Cross Hospital in Cairo.

Amongst the former sisters and nurses who have joined the Territorial Force Nursing Service are :—Sister Chatterley (assistant matron at the 1st Southern General Hospital, Birmingham, Dudley Road Section); Sisters Wheeler, Needham, A. Jones, M. James, K. N. Wooler, N. Jones, K. Worger, N. M. Sherwin; Nurses West, Stead, Campbell, Davidson, Haines, Elkins, Cameron, Peckham, Newnham, Radcliffe, Verney, C. F. Smith, G. Wright, Plumley, V. Townsend. Several of these are now working abroad. Miss F. M. Smith, who was trained at the Royal Infirmary and was sister there, has recently been appointed matron of the 2nd Southern General Hospital.

Bristol Royal Infirmary probationers are allowed to work for six months in the Royal Infirmary Section of the 2nd Southern General Hospital, and some fifteen are now nursing there. V.A.D. members have been received for instruction in the wards for short periods, and a limited number of special probationers have been received for training to fit them to serve as probationers in military hospitals. In addition to nursing many thousands of wounded soldiers, the infirmary has continued its civil work with small diminution of numbers, and has treated and lodged a number of wives and children of troops recalled from foreign stations. We record with much satisfaction that the decoration of the Royal Red Cross—First Class—has been bestowed on Miss A. B. Baillie, principal matron of the 2nd Southern General Hospital, Bristol, and matron of the Royal Infirmary, Bristol.

